# A multi-method exploration of a cardiac rehabilitation service delivered by registered Clinical Exercise Physiologists in the UK: key learnings for current and new services

**DOI:** 10.1186/s13102-024-00907-4

**Published:** 2024-06-08

**Authors:** Anthony Crozier, Lee E. Graves, Keith P. George, David Richardson, Louise Naylor, Daniel J. Green, Michael Rosenberg, Helen Jones

**Affiliations:** 1 Research institute for Sport and Exercise Sciences, Liverpool John Moores University, Byrom Street, Liverpool, L3 3AF UK; 2 School of Human and Behavioural Sciences, Bangor University, North Wales, UK; 3 School of Human Science (Exercise and Sport Science), The University of Western Australia, Crawley, WA Australia

**Keywords:** Exercise knowledge, Skills and competencies, Cardiac exercise provision, Research-based exercise prescription

## Abstract

**Background:**

Cardiac rehabilitation has been identified as having the most homogenous clinical exercise service structure in the United Kingdom (UK), but inconsistencies are evident in staff roles and qualifications within and across services. The recognition of Clinical Exercise Physiologists (CEPs) as a registered health professional in 2021 in the UK, provides a potential solution to standardise the cardiac rehabilitation workforce. This case study examined, in a purposefully selected cardiac exercise service that employed registered CEPs, (i) how staff knowledge, skills and competencies contribute to the provision of the service, (ii) how these components assist in creating effective service teams, and (iii) the existing challenges from staff and patient perspectives.

**Methods:**

A multi-method qualitative approach (inc., semi-structured interviews, observations, field notes and researcher reflections) was employed with the researcher immersed for 12-weeks within the service. The Consolidated Framework for Implementation Research was used as an overarching guide for data collection. Data derived from registered CEPs (*n* = 5), clinical nurse specialists (*n* = 2), dietitians (*n* = 1), service managers/leads (*n* = 2) and patients (*n* = 7) were thematically analysed.

**Results:**

Registered CEPs delivered innovative exercise prescription based on their training, continued professional development (CPD), academic qualifications and involvement in research studies as part of the service. Exposure to a wide multidisciplinary team (MDT) allowed skill and competency transfer in areas such as clinical assessments. Developing an effective behaviour change strategy was challenging with delivery of lifestyle information more effective during less formal conversations compared to timetabled education sessions.

**Conclusions:**

Registered CEPs have the specialist knowledge and skills to undertake and implement the latest evidence-based exercise prescription in a cardiac rehabilitation setting. An MDT service structure enables a more effective team upskilling through shared peer experiences, observations and collaborative working between healthcare professionals.

**Supplementary Information:**

The online version contains supplementary material available at 10.1186/s13102-024-00907-4.

## Background

In the UK, 26 million people live with a long-term health condition, of which 24% have two or more conditions [1]. By 2035 the UK population is estimated to grow by over four million, with a 50% increase in the over 65s, and a quadrupling of those with four or more illnesses (multi-morbidities) [2]. In recognition of this rise in both population growth and associated chronic and complex medical conditions, the National Health Service (NHS) long-term plan identified the need for clinical exercise services within acute care pathways to aid the prevention and treatment of non-communicable diseases [3]. The need for specialist exercise staff within clinical settings, primarily with higher education qualifications / backgrounds, has been frequently acknowledged [1–5]. Yet until recently, a lack of clarity has existed regarding what exercise services are being offered, to whom, and by whom to create an effective system-wide approach in exercise service provision for long-term health conditions [4, 5]. We [5] reported inconsistency in UK clinical exercise service provision, notably disparities in exercise specific job titles (e.g., clinical exercise physiologist (CEP) or exercise instructor) for individuals not part of statutory regulation, leading to inconsistency in staff scope of practice, knowledge, skills, competencies and experience within services [1, 2]. Such variances have led to a diverse workforce ranging from vocational to postgraduate master’s level qualified staff delivering exercise within clinical settings making it difficult to compare within and across services [1–5].

In the UK, clinical exercise service provision are most frequently available for those with cardiovascular disease (*n* = 242) or more specifically for coronary heart disease, with the British Association for Cardiovascular Prevention and Rehabilitation (BACPR) providing guidance to standardise exercise provision [2, 6] and the National Audit of Cardiac Rehabilitation (NACR) [7] auditing delivery. Although UK cardiac provision retains some similarities to its international peers (e.g., Australia) regarding service structure [8, 9], there are differences in staff knowledge, skills, competencies and job titles for those delivering the exercise components [2]. Indeed, structured education and employment pathways for registered/accredited CEPs have existed internationally across long-term conditions for ~ 30 years (e.g., Australia and USA) [10, 11]. Conversely, in the UK only 18% (*n* = 61) of exercise staff within cardiac services were postgraduate qualified CEPs [2]. This lack of consistency even in the most standardised service network is concerning when trying to regulate patient care and ensure patient safety [1, 2]. The recognition of Clinical Exercise Physiologists (CEPs) as a registered health professional in 2021 in the UK, provided a potential solution to standardise the cardiac rehabilitation workforce.

Our recent case study examining a unique, successful and large UK cancer pre/rehabilitation service found that exercise specialists were (typically) degree qualified and possessed equivalent knowledge, skills and competency levels to apply for Academy for Healthcare Sciences (AHCS) CEP registration via an equivalency process [12]. Yet, this level of qualification is rare as 88% of exercise delivering staff in UK cancer services did not possess an undergraduate degree or higher [2]. Therefore, a purposeful case study to understand how registered CEP knowledge, skills and competencies contribute to the provision of a cardiac-based clinical exercise service is valuable to understand best practice, as job titles alone are not sufficient to judge service effectiveness or staff qualities [1–3, 5]. Consequently, the service purposefully selected to be examined in this study was chosen because it was; (i) well established (*n* = 30 years) and delivered clinical exercise provision by registered CEPs as part of a multidisciplinary team (MDT), (ii) delivering exercise to wider range of patients with cardiovascular disease including high-risk cardiac and vascular conditions as well as with congestive heart failure, (iii) conducting research into enhancing exercise service provision for cardiac rehabilitation as well as other conditions, (iv) uniquely operating in a dedicated building for exercise services with use of a purpose build gymnasium for strength and conditioning, and (v) commissioned by the NHS. This purpose-built study aimed to explore how CEP staff knowledge, skills and competencies contribute to the provision of a cardiac-based clinical exercise service, how these components assist in creating effective service teams, how they differ to previously explored services, and to identify what challenges currently exist from staff and patient perspectives.

## Method

### Design and theoretical underpinning

#### Design and theoretical underpinning

A case study format employed ethnographic principles (the exploration of peoples’ habits and beliefs) to uncover values and attitudes retained by the participants [13]. Multiple qualitative methods (online semi-structured interviews and face-to-face observation and field notes) were employed to explore the service from staff and patient perspectives both individually and collectively [13]. This qualitative multi-method approach, combined with the longevity of the study and data triangulation, was employed to reduce potential social desirability and bias from staff and patient perspectives [14]. Ethical approval was obtained from East Midlands - Leicester South Research Ethics Committee [ref: 21/EM/0227]. The lead researcher spent 2–3 days per week for 12 weeks in the service between April - July 2022. The ethnographic data via field notes and observation was the primary focus allowing rapport to be developed with both staff and patients before the completion of semi-structured interviews at the end of the 12 weeks [14].

#### Consolidated framework for implementation research (CFIR)

A comprehensive implementation framework (CFIR) was adopted [15]. CFIR links existing theories to create ideas concerning what works, where, and why within services, aiding future service implementation and evaluation [15]. Specific components relating to service delivery including staffing structures, staff skills and competencies, and patient perceptions allow a detailed exploration of these areas through contextual discussions regarding service operations [15, 16]. All five sections of CFIR were drawn upon throughout this study (see Table 1) and provide a framework for interview guide (additional file 1).

**Table 1:** Consolidated framework for implementation research domains and constructs applied across each research study [14]

CFIR Domain	CFIR construct to consider	Overarching context within the case study
**Intervention development & challenges**	**Intervention design & evidence**	Interventions have ‘core components’ (the essential and indispensable elements of the intervention such as the exercise delivery) and an ‘adaptable periphery’ (adaptable elements such as exercise locations). This domain focused on how the service was designed and operated.
**Service users and resources**	**Economic climate and Patient needs**	Changes within the outer setting, such as service funding can impact how the service will proceed with its offering. This domain focused on the barriers faced by service users and what resources were available to support them.
**Organisation & structures**	**Service characteristics**	This domain focuses on how the structure of the service (staffing, age, size, qualifications) impacted the implementation of the intervention.
**Staff skills & perspectives**	**Staff Knowledge, skills, competencies and beliefs**	This domain focused on the individuals within the services (primarily exercise specialists) and how their cultural, organizational, professional, and individual mindsets and beliefs impacted service provision.
**Service process and effectiveness**	**Staff beliefs regarding effectiveness**	This domain focused on service effectiveness (or not) and the key indicators of it from staff perspectives.

### The AHCS registered CEP-led cardiac service

This NHS service was created over 30 years ago, initially as a nurse and physiotherapist-led cardiac rehabilitation programme, which shifted to being CEP-led for exercise provision ~ 25 years. The programme is delivered over two sites. The primary site being community-based, the other being within a hospital. An umbrella term for the service is cardiac rehabilitation, yet face-to-face exercise support for patients is offered for a variety of cardiac (e.g., post-myocardial infarction), vascular (e.g., peripheral vascular disease) and heart failure (e.g., left ventricular failure) conditions. Patients are contacted after diagnosis or treatment (either surgical or non-surgical) regarding the uptake of physical (e.g., exercise), nutritional and psychological support. Full details of the intervention are provided in Table 2 [17].

**Table 2:** Intervention components mapped onto items 1 to 9 of the template for intervention description and replication (TIDieR) checklist

Item Number Item	Item
**­­­­­­­­­­­­­­­­­­­­Brief Name**	
**1**	Cardiac rehabilitation
**Why?**	
**2**	Cardiac rehabilitation for patients undergoing treatment for cardiac (post-myocardial infarction) vascular (peripheral vascular disease) or heart failure (left ventricular failure) conditions in the Midlands, UK.
**What?**	
**3**	**Intervention resources **
	**Fitness: **Equipment available was gym-based machinery such as cardiovascular (Ski Erg, rower, treadmills, bike, Cross Trainers) and resistance machines (chest press, leg press, seated row, shoulder press), in addition to free weights (dumbbells), medicine balls, TRX and resistance bands (various resistances). Assessments were carried out using ergoline bikes, ECGs, blood pressure monitors, oxygen saturation monitors, weight and height scales, a hand grip machine and shuttle walk cones. Home-based exercise programmes were available to service users.
	**Nutrition:** Referral to dieticians were made as part of the MDT support system.Clinical Nurse Specialists: Managed the service user caseloads in conjunction with CEPs, providing educational support for behaviour change at various stages of the intervention.
	**Clinical Nurse Specialists: **Managed the service user caseloads in conjunction with CEPs, providing educational support for behaviour change at various stages of the intervention.
4	**Procedures and key components **
	Referral pathways were developed based on cardiac, vascular or heart failure patient status. Referral forms were completed and electronically processed via the administration team of the service.
	**Practical application:** A variety of physical assessments are conducted, primarily an exercise tolerance test via a bike or treadmill with a RAMP protocol will be completed with an ECG attached. Other measures may include:
	**Physiological testing:** 6 Minute Walk Test, Incremental shuttle walk, Hand grip, Sit to stand.
	**Health Measures:** Blood pressure, Resting Heart Rate, active heart rate, Blood oxygen saturation levels, Height, Weight, Medical history
	**Questionnaires:** Lifestyle questionnaires that could be used are: International Physical Activity Questionnaire, EQ-5D quality of life, Stages of change/readiness to change
	**Eligibility:** Anyone with a cardiac-related (umbrella term) diagnosis
**Who will provide?**	
**5.**	The service is provided by the NHS. Various referral pathways were used:
	**Referring health professionals:** Referrals were accepted from all health professionals (Consultants, GP’s).
	*Staff in service at the time of the study: Band 8 Clinical manager (n=1), Band 7 Service Manager (n=1), Band 7 CEP service lead (n=1), Band 5 (n=1), band 6 (n=2) and Band 7 (n=1) CEPs, Band 6 Clinical Nurse Specialists (n=2), Band 6 Dietician (n=1).
	*Note 1 x Band 5 CEP has since been employed and did not take part in the study.
	**A full staff structure can be seen below:**
**How?**	
**6.**	Registered CEP-led: Face-to-face consultations and group exercise format delivery. Previously, physiotherapy and nurse-led but changed due to a combination of physiotherapy availability or lack of, and increased conversations with members of BACPR who advocated the use of exercise specialists within clinical exercise services.
**Where? **	
**7. **	All service user consultations and activities took place face-to-face on site at either hospital or community locations.
**When and how much?**	
**8. **	The intervention had a 12-week funded period of rehabilitation. The programme was restricted in terms of days/times service users could use facilities with two sessions per week allocated based on patient choosing. Once 12-weeks had been completed service users had the option of continuing to attend the facilities at a subsided rate of membership under the supervision of privately employed registered CEPs.
**Tailoring**	
**9. **	All sessions were tailored to individual goals and used individualised exercise prescription developed by registered CEPs and based on exercise assessments.

### Participant recruitment

Participant recruitment was based on convenience sampling across both staff and patients, with all CEPs expressing a willingness to participate. An initial (virtual) scoping meeting was conducted with the service multidisciplinary team (MDT) [17]. In this service the MDT included AHCS-registered CEPs, dieticians, cardiac nurse specialists, and clinical service leads/managers, with occasional consultant interactions in the event of unforeseen complications. The meeting explained the study aims and objectives, after which written consent was obtained covering each aspect of the data collection, including semi-structured interviews and observation. The final sample included MDT staff (*n* = 10); a clinical service manager (*n* = 1), clinical service lead (*n* = 1) who oversaw the intervention, AHCS-registered CEPs (*n* = 5), cardiac nurse specialists (*n* = 2) and a dietician (*n* = 1). Staff members were white British, female (*n* = 7) and male (*n* = 2) and black male (*n* = 1), aged between 26 and 45 (mean age of 40). All participants were employed full-time by the NHS with a minimum of two years’ experience in the role.

Patient recruitment was conducted using a verbal announcement before weekly classes (*n* = 8) asking if attendees (*n* = 45) wanted to participate in the study with field notes used to record observational data, including conversations. All patients attending the sessions verbally consented to observational data collection by the lead researcher and were provided with a written study information sheet and consent form. No one formally declined or stated any reasons for not taking part, but often participants preferred to concentrate on the exercise components without fielding questions during conversations for data purposes. Patients (*n* = 7) were white British, female (*n* = 3), male (*n* = 2) and Asian, male (*n* = 2). Patients were retired/not working, had a mean age of 61 years, reported various long-term medical conditions, but were specifically referred due to having one of the umbrella terms of cardiac-related conditions accepted via the service inclusion criteria (e.g., post-myocardial infarction, heart failure, peripheral vascular disease). The research team’s involvement was limited to participant recruitment and data collection.

### Data collection

Staff participants had individual caseloads, although patients came together during group exercise sessions. Patients were assigned to specific sessions (days/times) of their choosing, yet could be unpredictable in their attendance due to various factors (e.g., health, transport). Observational data in the form of field notes were used to capture a sufficient cross-section of patient experiences across different sessions within the intervention, encouraging peer interaction and the promotion of shared experience where possible [12].

### Semi-structured interviews

The semi-structured interview guide (see additional file 1) was developed based on the CFIR framework. Pilot interviews were conducted by the first author with three independent researcher peers prior to study commencement to enhance credibility and refine interview questions where necessary [12]. Interviews (*n* = 10) were conducted on an individual basis by the first author via a secure virtual platform (Microsoft teams) lasting 28 min on average (ranging from 24 min to 36 min). Written consent was obtained and interviews were visually and audio recorded with prompts and probes used to elicit more detailed responses from participants [18]. A brief verbal summary was provided by the researcher at the end of the interview to clarify the main points and allow participants to add further information (where required) [19].

### Observation and field notes

Ethnographic principles were adopted during the observation of the setting, including the daily practices of the staff and their patient interactions [13]. Notable moments were written down in a note pad in the form of keyword entries [14]. Memories and reminders in the field notes then allowed the observations and conversations to be developed into a research log, typically completed during lunch breaks or at the end of each day, and never more than 24 h after the original observation to prevent the risk of memory fading and details being lost [14, 20]. Such accounts were accompanied by researcher insights and interpretations of events which contributed to the understanding of the setting and a narrowing of the research lens [14, 20]. During this process, the research team acted as “critical friends” and theoretical sounding boards [21].

#### Data analysis

Data obtained through the semi-structured interviews and field notes via participant observations were audio and visually recorded using a portable Dictaphone and Microsoft Teams, then transcribed verbatim. Data were thematically analysed manually using reflexive thematic analysis recommendations such as data familiarisation, generating initial themes, coding and finalising patterns of shared meanings underpinned by a central concept, and writing up using data extracts interspersed with researcher insights and interpretations [22]. Although the data themes generated were (deductively) linked in relevance to the pre-determined categories formed by the CFIR-guided research questions, the patterns of shared meanings were generated from the data themselves allowing interpretation and researcher contextual awareness to be discussed [22]. Flexibility in analysis was driven by both the prevalence (number of speakers articulating the theme) and the importance placed on information [22]. It is important to note that “data saturation” or “data adequacy” could be assumed as no new themes were identified when analysing the final few transcripts [23, 24]. Primary analysis was conducted by the first author with frequent debriefing sessions with the research team to discuss, challenge and reframe the thematic structure [21, 25].

### Creating the non-fiction composite characters

Large volumes of data were collected and analysed, therefore, alongside confidentiality issues, it was deemed unrealistic to present singular case studies for all staff and patients [26]. Subsequently, four ‘composite characters’ were created to tell the stories and journeys throughout the service. The narratives of the four participants were created based on participants who shared *some* similar, common experiences or backgrounds during their time within the clinical exercise service setting, but also have potentially different perspectives on the culture of clinical exercise services [26]. The theme and identity that holds these characters together are; Character 1 (Sam) was a CEP with more than six years’ experience in the role, undergraduate degree qualified in sport and exercise science with additional vocational qualifications in cardiac rehabilitation; Character 2 (Lauren) was a CEP with a minimum of three years’ experience in the role, has a master`s degree in a clinical exercise physiology-related field and additional vocational qualifications in cardiac rehabilitation; Character 3 (Tom) represented the wider MDT team of non-CEP clinical leads/managers, clinical nurse specialists and dieticians who had undergraduate degrees, Health and Care Professions Council (HCPC) or relevant nursing council registration and excess of five years’ experience working in cardiac rehabilitation; Character 4 (Mira) was a retired/non-working patient attending the 12-week programme due to cardiac-related condition. The stories and interactions are told using the CFIR themes as underpinning headings, through the critical moments that occurred within the journey through the programme, but not necessarily in chronological order [27]. The composite character interactions are told from the researchers first-person perspective as they had come to understand them [28].

### Lead researcher positioning

Given this study was based upon ethnographic principles, lead researcher self-reflexivity was important due to researcher background and training within clinical exercise provision [29]. This experience could provide pre-conceived ideas regarding exercise provision and enable a broader interpretation of participant concerns or thoughts [29]. Such reflection means that this article will retain the use of “I,” “me,” or “my” on occasion and as such refers to the first author [29]. What follows is a researcher’s story of “self” experience, alongside the “other,” in this case, the collective thoughts of staff and patients concerning their experiences within a clinical exercise service [29, 30]. The data extracts represent each individual’s experiences and opinions at a given time and, in combination with my observations, re-creates a holistic view of experience representative of what any individual may be exposed to in the service at a point in time [29, 30].

As an AHCS registered CEP who had been employed in a similar role previously, I acknowledged that I needed to see past my own preconceptions or bias and use such constructs as a basis to probe further into specific actions and behaviours. An open and honest relationship developed during the early weeks of observation with any researcher vs. participant barriers seemingly lowered after the initial 2–3 weeks. At the outset I would have classified myself as an outsider in collaboration with insiders given that I approached the service to observe it [31, 32]. Yet, after this initial period and given my cardiac rehabilitation background, the relationship felt like it had morphed into one of an insider, in collaboration with other insiders due to the flowing nature of the conversations and the mutual respect that appeared to develop through shared experiences [31, 32]. The following findings and discussion include reflective extracts that are in italics, indented and single-spaced to ensure separation from the descriptive representation.

## Results and discussion

### Introduction

The service was exiting COVID-19 restrictions when I was first introduced to Sam, Lauren and Tom, resulting in a pre-arranged virtual discussion over MS Teams. At this point they had little knowledge of me, my background, or how I might portray them and their service. After a brief introduction I verbally explained my purpose. Following this, I paused to allow time for questions/concerns (of which few were raised). I felt my research aims were understood and an acceptance of me (given my background) was initiated. Although the online meeting was challenging as it was hard to gain a true representation and sense of feeling displayed by staff, I had experienced this before (e.g [33]) and was prepared for some silences [34]. Moreover, because all staff were engaged (currently or previously) in research projects within their work, there was an understanding of what to expect and a recognition that research is vital in furthering the evidence base and maintaining currency in the field. Their acknowledgement of, and familiarity with, research alongside their level of comfort with observation was reassuring. I would be surprised if other services without research links would have been so at ease. I left the meeting feeling content that I had set the tone for my face-to-face encounter in a few days.

### Face-to-face contact

I arrived on the primary community-based site early hoping to create a good impression, but also expecting to see how staff prepared for the day ahead. I received a warm welcome with open body language while being re-introduced to the team by Sam. A walk around the facility followed, accompanied by an explanation of the current staffing levels (two CEPs had recently left) and how that impacted exercise sessions. The building was two-floored, on the bottom was a café and seating area for patients to relax, prepare and recover from their exercise sessions. It had toilets, changing and showering facilities and included the main gym floor area where the cardiac-based sessions took place. The first-floor featured meeting rooms, offices, assessment rooms and exercise studio space which contained portable equipment for use within classes when applicable. The first hint of NHS involvement and clinical working was the separation of these spaces. There was a clear divide from a logistical perspective; keypad restrictions were in place throughout the second floor to negate public access, alongside telecom access through the front door into the building itself. The gym environment had a friendly, yet clinical feel, mainly due to uniforms displaying NHS logos, half of which were worn by clinical nurse specialists. Mask wearing by staff, although no longer mandatory by law, provided another example of how (inadvertently I`m sure) the service presented a clinical feel. Yet its size (roughly 20 m by 15 m) and the volume of apparatus (six rowers, two Ski Ergs, double digit treadmills/X-trainers, resistance machines, TRX suspension trainers, free weights and portable equipment) made the gym unique in its appearance compared to other clinical services I had observed.

*This was a considered layout that maximises the available space. An area for walking around the equipment was ideal for a warm up and cool down and guidance resources were on the walls (information that could remind patients about how they should be feeling such as Rate of Perceived Exertion (RPE) and claudication charts, stretching and resistance-based exercise posters). Anecdotally this is not uncommon, but I`d be interested to see how staff use these – do they get patients to actively engage with the materials? Or, are they included because it`s best practice only?*

Sam and I discussed current service operations, but swiftly digressed into how incorporating virtual exercise classes could improve their offering. There was a feeling from Sam that integration of online sessions could potentially lessen some of the access barriers regularly cited by patients (e.g., transport), in addition to advancing their `menu-based` delivery [35]. Rather unexpectedly, this discussion shifted into a Q&A led by Sam who wanted to understand my experiences of virtual exercise delivery (an area I had previously observed, e.g [33]).  Offsetting safety with expanding reach and exercise adherence were main discussion points, but from a personal perspective being a sounding board for virtual exercise delivery in practice felt fantastic and demonstrated a level of acceptance even at such an early stage of my stay.

*One of my underlying concerns before I entered the service was the staff perceptions of me, as an unknown entity I expected doubts about my skillset and knowledge of the setting. It was refreshing to openly chat about patient screening and risk assessment, accessibility, exercise prescription and adherence – all of which I was familiar with and could offer insight into. My impression was that Sam gained confidence in my ability during our conversations (shared knowledge and findings) which demonstrated my own researcher and practitioner credibility. Over time I found these types of discussion became more prevalent and enabled me to be accepted as a peer rather than seen as an outsider collecting research. My opinions mattered and a more natural relationship with the staff began to form.*

#### CFIR Sect. 1.1 – CEP-led service conception

I entered the service knowing that certain components were unique compared to the wider cardiac rehabilitation landscape through my previous research; primarily the sole use of registered CEPs for exercise delivery, compared to unregistered CEPs or exercise specialists with vocational qualifications [2]. I wanted to understand the reasons behind this; Did it change, why and how long has it been like this? Numerous conversations skirted around the subject over the weeks as both Sam and Lauren acknowledged that it was all they had known within this service. Interestingly, they talked about their initial assumptions regarding exercise delivery within clinical services, which centered on the belief that others (services) followed suit and utilized CEPs similar to themselves with training and education in exercise prescription and the ability to become registered once AHCS registration became available;

*Sam: “I`ve never known anything different, it`s only when you start talking to other people (at other services) that you discover they are different…lots (of services) are nurse or physio-led…I don`t know why or when we changed…but to me it comes down to the knowledge and skillset…exercise prescription quality and knowledge…I feel the CEP background of exercise prescription is strongest due to CEP degree training (undergraduate and/or postgraduate)…physio`s and nurses look at things in a different way, more recovery focused”.*

Without wanting to misrepresent the abilities of other health professionals, Sam made it clear that CEPs were his choice to lead the exercise component within clinical services. CEP inception within this service, however, came from somewhere and it was only during a conversation with Tom at the back end of my stay where I finally found some answers;

*Tom: “We were working with physiotherapists some ~ 25 years ago, but they were coming to the end of their careers, so we looked at what was going on in America, how their private care providers worked. At the time BACPR was just taking off, we did some exercise-specific training for the nurses and we were approached to see if we wanted to take on a very young exercise specialist (officially titled a CEP) for 7 hours a week to complete our assessments and it grew from there…it was a new concept, nationally rehab was more about the nursing teams and physiotherapists… overall care could be very static and traditional for these patients…discharged at 10 days…rehab was 6–8 weeks post-MI but (exercise) with the physiotherapist felt quite static…a one size fits all approach…we felt that having a CEP that was solely exercise focused would be beneficial”.*

*This was one of many lightbulb moments for me. This service did not want to just follow tradition. Although they had no issue with the exercise provision that was being provided and valued the work of the current team (physiotherapists), they wanted to explore additional ways to improve the service and enhance the care they offered…they felt that a combination of healthcare professionals would do this and expanded the skillset across the team.*

Tom identified that personalized patient care had to be at the forefront of service delivery. Exercise, although prominent in later stage care, was now even more vital in the rehabilitation process, therefore, the most specialist people (CEPs) were needed to deliver it.

#### CFIR Sect. 1.2 – referral pathways and health care professional interactions

It was clear from the outset that this service had a well-established referral pathway due to the levels of organization and clear protocols that were in place (e.g., referral forms sent via secure NHS email). Lauren explained that *“Patients are picked up on the wards by the nurses, they’re referred straight into the service, from bedside risk factor education to consent for exercise participation”.* From a patient perspective, the ease of the journey was vital. Mira acknowledged that the service was efficient; *“I couldn`t believe how quick I got started and how thorough it was…they helped me understand my condition and that it was safe to exercise”.* It became apparent when discussing referrals that the ability to educate and `recruit` patients efficiently was, in part, explained by their capacity to engage straight away, but also the knowledge and skills of how to communicate with patients. Conversations were engaging, open and friendly, with active listening taking precedence. I observed Sam discuss Mira`s procedure (angioplasty) and then listened as he provided a detailed brief of the steps she would go through during her time in the service, allowing questions along the way.

Active participation in research was also vital in the development and exploration of different referral pathways. One example being the Post-sternotomy Cardiac Rehabilitation Exercise Training Study (SCAR) which identified that (qualifying) patients could be exercising earlier than guidelines stated (2 weeks rather than 6 weeks post-sternotomy) [36]. This innovative research was translated into practice and shifted the referral process guidelines within this service into a new evidence-based format;

*Sam: “I worked on the SCAR trial which monitored patients during exercise earlier than normal post-surgery…6 weeks was the guidance, but that had seemed to be plucked out of thin air with no real evidence…once the study had finished I went to the surgeons at the hospital, delivered the outcome evidence and developed new referral pathways…we have started bringing people in earlier, I had someone today who is at 4 1/2 weeks, rather than them sitting at home festering for 6 weeks”.*

Generally, exercise services will rightly follow national guidelines (e.g., BACPR) for patient recruitment, this service (due to the knowledge and skills of the CEPs) looked to use their latest evidence to enhance patient outcomes in areas such as cardiovascular fitness in an earlier timeframe [36]. Moreover, this decisive integration into the rehabilitation system allowed a speedier onward referral into the CEP-led phase IV service that was available on site but delivered by a private partner. The concept of phase IV is nothing new, but having CEPs deliver it is quite unique [2] as Sam explained to me;

*“It`s great being able to see patients move from us (to phase IV) and remain here…we can follow their journey from a distance…it`s great to know they`re remaining active…being looked after by similarly qualified CEPs in phase IV…I don`t think this continuity of care is common”.*

*Having that seamless onward referral process extremely positive for the service. There was a level of trust in Sam`s voice, happiness in the knowledge that patients were going to be looked after by equally qualified peers.*

An on-site MDT was a positive factor in the whole referral process (more control and capacity to deal with the patient flow) [37]. From working in this setting, I know that creating a fluid, timely and consistent patient journey is not easy. Here, in part, it came from longevity of the service (pathway development over time), but primarily through the knowledge of how a clinical service should operate and a willingness to implement it by the team. The service had long-standing relationships with the hospital-based health care professionals (e.g., consultants) which enhanced their referral pathways. Even though patient suitability and uptake fell within the realms of Tom, frequent conversations by all team members could be had with consultants regarding surgical or non-surgical treatments, complications, or re-referrals if contraindications were identified at any point. Yet, even in this service, Sam made me realise that interactions with health care professionals can, on occasion, be difficult and sometimes a barrier to providing specialist care;

*Sam: “…we speak to consultants, there was a time when they were not really sure who I was or what I did, for example, if I noticed someone hadn’t been started on a medication and queried it, they’d wonder why as I was not a nurse…I think it was a lack of understanding…it’s a fairly new role and not many trusts have CEPs”.*

*This lack of awareness, even in such an innovative service, was concerning, but not uncommon based on my own experiences. Sat in the office during admin time, I wanted to understand the cause of this issue so I questioned the team;*

*AC: “How could you improve your relationship with other health professionals?”*

*Sam: “…recognition of our role within a hospital setting in terms of registration is one way, but it`s challenging, we’re not recognised as a as an allied health professional, even though we`re now AHCS-CEPs”.*

*AC: “How`s that a problem?”*

*Sam: “It’s the understanding of our role, people (in the NHS) don’t feel that we have the skillset to deliver the intervention, sometimes it can be an issue, I`m sure it could impact referrals (in other services)”*.

AC: “*But it doesn’t here*?”

*Sam: “For us, not massively, but I would imagine it could do if we were not as well established as we are”.*

On a wider scale, referral pathways could be impacted if a lack of confidence from referring practitioners were identified [4]. Moreover, perceptions about scope of practice may impact the patient journey, and ultimately the level of care they receive [4].

*After two months of observation it is evident and acknowledged by the team that service links with health care professionals were in place. Having Tom on the wards with a nursing background made the whole process easier. Perceived or real scepticism from other health care professionals in NHS regarding the skillset and scope of CEPs was concerning. The questioning of their belonging and what they could offer, especially as they now hold healthcare registration, demonstrates that an awareness of CEPs is needed to improve interprofessional relationships which can only improve healthcare services in the long-term.*

#### CFIR Sect. 2.1 – patient integration and support

A few weeks into my observations and keen to see the level of support Mira received, I sat in on an outpatient consultation (on site in the community exercise facility) led by Tom. This was the first time Mira met Tom outside of the hospital environment (4-weeks post-surgery). We sat in a small office upstairs which reminded me of a doctor’s surgery with its white walls and randomly placed NHS-based posters. There was a relaxed atmosphere yet the discussion, although polite, friendly and occasionally humorous, was clearly one of a patient/nurse due to its interview-based format. Tom and Mira exchanged high volumes of questions/answers/explanations, with the occasional probing for additional information by Tom. Mira described the build-up to the heart attack; “*…I had shortness of breath on various occasions when we were walking…it led to some chest pain…my husband called 999”.*

Throughout the conversation Tom was friendly, clinically focused, yet compassionate during the enquiries, empathy was evident and matched by a clear understanding of the experience Mira had undergone. It was fascinating (and not uncommon I`m sure) to hear Mira describe the discomfort as *“coming out of nowhere”*, an interesting observation given the discomfort occurred at *“multiple times”* leading up to what was eventually diagnosed as a heart attack. Tom educated Mira that her symptoms were signs of a heart attack and not uncommon, frequently using lay terminology to explain the complexity of the condition and associated surgery via visual and verbal descriptors (e.g., pictures). This demonstrated a high level of knowledge, skill and experience as it factored in patient learning styles to the information delivery. In addition to re-enacting the sequence of events, this consultation was used as an extension of the behaviour change discussions from Mira`s bedside (e.g., risk factors for future events). The conversation shifted towards medications as Tom described (in great depth) each tablet Mira was taking, its purpose, side effects and why it was important.

*I would have expected medications to have been discussed earlier, only to discover (via Tom after the consultation) that it had been, but to enable adherence there was a constant reiteration in the importance of compliance. This in itself formed a major part of Mira`s lifestyle change, one that had been thrust upon her quickly. I got the impression that without this discussion, medications could have been seen as short-term and not necessary, just from her body language and terminology used.*

Mira could easily have fallen back into a curative mindset, no longer associating risk of future events with medication conformity. This again was a teachable moment created by Tom and relayed in a manner that Mira appreciated and hopefully accepted. A referral for CEP assessment was explained and consented, with Mira extremely receptive to attending. Overall, the appointment lasted ~ 45 min, not especially drawn out by either party, so I would take this as a standard timeframe. This consultation confirmed that support was individualised and tailored to Mira. Moreover, it continued the theme of a seamless patient journey created by a diverse MDT working efficiently in conjunction with their service protocols [37].

*The behaviour change element was definitely initiated then followed through by Tom. Long-term observations (12 weeks) showed that it was Tom who began the goal setting process and CEPs only got involved during the exercise sessions when trying to encourage patients to work in a range of intensity or duration that facilitated progression. Behaviour change was not something the CEPs touched on in great detail, in fact, they themselves recognised it as an area they needed to improve and they had undergone a change recently to try an address the balance…*.

I arrived on site (during week 8) to find no exercise sessions were planned. Sam explained that a behaviour change/education session was taking place;

*“…we`ve tried the traditional exercise followed by education sessions that most services use…these were ok but some (patients) weren`t in favour…we now tell people there is no exercise this week and to turn up for some talks about how to manage their condition, but uptake hasn`t been great and the feedback is that they`d prefer to be exercising”*.

The session I witnessed was led by Lauren via a Microsoft PowerPoint presentation. The group was small, I got the impression (closed body language through crossed arms) that they really wanted to be exercising rather than talking about cardiac risk factors, in fact, there was little engagement in the talk, and they finished early. Interestingly, Mira asked if they could use the gym before they left. After the talk Lauren gave her opinion on how it went; *“It`s hard to find a balance, the turnout was disappointing (only 4 people rather than the 12–15 in sessions normally) and lack of interaction made it hard for me”.* During the talk Mira frequently nodded, acknowledged information and appeared to understand the content, yet was not willing to step outside of the self-created comfort zone and answer questions that were posed or even challenge ideas that were presented, either positively or negatively. Was this due to the lack of numbers, or just the nature of the situation, i.e., discussing personal trauma in front of others, even those experiencing similar circumstances can be daunting and intrusive? Mira`s passion to exercise however was clear;

*“…To be honest I`ve come because I feel that I should, the team are great and I don`t want to let anyone down, but really I`d prefer to be downstairs (exercising)…I can read this in a booklet I got off Tom anytime”.*

There seemed to be a lack of value associated to the education (Mira`s perspective), suggesting that a format change may be needed for future cohorts.

*That night I thought about behaviour change within services, especially what I had experienced and seen over the past few weeks. Was it ineffective or was it just wrong place/time in this service? Staff here were excellent at delivering impromptu support during conversations. Moreover, the patients identified the CEPs as exercise specialists, this is what they wanted from them, to get `fitter`. But, if the CEPs continued to subtly use their communication skills to integrate more behaviour change prompts into simple conversations, it may be sufficient. Sam and Lauren had the skills; the communication was good, specifically their empathy and active listening as they took note and responded to Mira when required. What this service lacked was a clear strategy, but not through a lack of trying or the ability to deliver the content successfully. A generic consideration may be how/when services present behaviour change information to a patient, who does it, and the depth of information needed.*

#### CFIR Sect. 2.2 – patient safety mechanisms – `the huddle`

Patient safety underpinned everything I witnessed within this service. One of the most prevalent and enlightening examples of this was the `team huddle`, a daily activity that included the whole MDT. This event was equivalent to a pre-exercise session meeting taking place in a small conference room, whereby all patients were discussed re: progress and status (new starters and current attendees) including condition overviews and adherence. New starter referrals were explained in detail by either Sam or Lauren to ensure everyone who had contact with them were aware of any considerations such as medications or multi-morbidities. Sam/Lauren, in conjunction with Tom, completed the fitness assessment and retained primary responsibility and case management of specific patients during the exercise component, yet all staff were required to monitor the sessions and therefore needed these updates. On a Friday the discussions included a summary of the past week and information about the forthcoming week, including session fill rates, fitness testing waiting lists and any issues or potential problems (e.g., service capacity due to staffing levels or holidays). The shared responsibility and addition of Tom into these discussions highlighted the integrated nature of the service;

*Tom: “There is nothing better than sitting and listening to the team discuss patients…there`s a real crossover of skills and learning via the shared experiences…everybody’s upskilling without even knowing it”.*

This collective and unified working process is an unofficial and unaccredited knowledge exchange that enhances MDT skillsets [37]. The huddle facilitated this learning. Allowing different members of the team to lead the huddle each day fostered personal growth, developed workplace craft and enhanced the team ethic, demonstrating that each member held equal status concerning patient safety and were capable of adhering to NHS policy in this area [37, 38].

*Staff were vocal, no one hid, which demonstrated solidarity and respect – no fear of being chastised if they spoke. In the past hierarchal status might have overridden everything else, but this discussion was informal with room for social banter if the opportunity arose. Kudos regarding any achievements were given, but at the same time areas for improvement and development were highlighted. I watched an inclusive and engaging 15–20 min `chat` each day which created a learning environment in patient centered care, something I`d have liked to have been part of in the workplace.*

#### CFIR Sect. 2.3 – patient safety mechanisms – fitness assessments

Patient facing activities that required a high level of risk management and in-depth safety protocols were often completed jointly between Sam/Lauren and Tom, an example being fitness assessments. Clinical services utilize a variety of fitness assessments, some highly clinical (e.g., cardiopulmonary exercise testing) and others more field-based (e.g., 6-minute walk test) [39]. The primary one in this service was a submaximal bike or treadmill assessment with a 12-lead electrocardiogram (ECG) and blood pressure monitor attached to the patient during the assessment. I observed Tom and Lauren work in combination to monitor Mira during a bike assessment. Lauren used lay terminology to explain the procedure, including the function and purpose of the ECG and focusing on what Mira would be asked as she pedaled (such as RPE levels). Mira seemed slightly anxious as Tom attached the leads, uncertain of what lay ahead, but Tom was empathetic whilst explaining how the results would help Mira`s exercise programme design. Mira was happy with this and continued though the assessment, pedaling at the required speeds against the increasing resistance and answering Lauren`s questions regarding RPE levels, while Tom monitored any ECG changes. The assessment itself went without issue. Mira looked comfortable throughout, even when faced with increasing resistance she challenged herself, clearly understanding the importance of providing an accurate representation of her capability as was explained by Lauren and Tom before she started. This itself displayed a high level of communication skill, specifically empathy as Mira was anxious about the unknown, yet this was managed by Lauren using active listening and questioning to dispel any undue fears. After Mira had left, I questioned Lauren and Tom about the importance of the assessment process in relation to patient safety;

*Lauren: “Assessments are really important…it’s the first time we see that patient from an exercise viewpoint…understanding their fitness levels and their physiological responses is vital…observing the ECG, detecting issues…you need to be competent by conducting the assessment correctly, but also interpreting that information, then developing an exercise prescription that is fit for purpose”.*

Lauren recognized that patient safety is multi-dimensional. Not only is there a theoretical knowledge requirement of how to carry out the assessment, there is the skill of completing it safely whilst screening/monitoring patients and then competently analyzing the results to formulate suitable exercise prescription. Tom outlined the nurse role;

*“…I`m responsible for recognition analysis; having an understanding of ECG, chest pain management, the safety aspects for patients that have had a sternotomy during an assessment, aetiology of any particular condition and what adverse reactions we could see…we monitor that in conjunction with the CEP, it`s a shared responsibility, the CEP takes the lead but we collaborate throughout”.*

It was interesting to hear that this service had adopted a policy of using both CEPs and clinical nurse specialists within the assessment process to further reduce risk. This `belts and braces` approach seemed to be valued in this service. It was good to see this level of collaboration between staff as I doubt a newly qualified, inexperienced CEP, with minimal exposure to a real-world setting would have been able to undertake that assessment safely and effectively without it. Personally, I think only being exposed to this type of situation would prepare you, therefore, having work placements during your training would greatly increase your understanding of the standards needed to provide safe, patient-centered care in a workplace, thus raising the standard of CEPs coming out of education settings.

#### CFIR Sect. 3.1 – MDT roles: training and development

Internal training and development have been recognised as good practice within MDTs [37, 38] and it was pleasing to hear that this was evident in this service;

*Sam: “I came here for work experience and basically stayed…the varying types of experience was great, you had people that had worked here for over 10 years…there were loads of opportunities to learn from others in the team, being able to sit and observe… discussing how and why they worked that way”.*

As Sam moved quickly to assist Mira with the rower set up, I pondered about how this type of learning or craft within a real-world setting can only be achieved with the support of highly trained and skilled peers [38]. Moreover, a few weeks later the subject of planned supervision and observation was raised again by Tom;

*“…CEPs come out of university with a masters or BACPR qualifications and that’s great, but it`s less clinical and includes less placement time (if any in non AHCS accredited masters courses) than a nursing degree… it’s that hands on experience that`s really important and where the learning occurs, and that`s missing”.*

Tom identified that work placements are the cornerstone of a nursing degree, and this type of experience cannot be overlooked for CEPs. Here, internal staff training included observation of all MDT roles during the first few weeks of employment. This promoted growth for all staff, i.e., leadership opportunities for more senior members of the team through unofficial mentoring, and theoretical learning and practical application experience for the newer members of the team. Tom stressed that staff development was vital for preserving a consistent level of provision (and staff engagement/retainment) within the service. Moreover, the research generated by the team added further knowledge and skill development as described by Sam; “*…completing research with the university brings a whole new light on rehab as a service…our learning and how we think about exercise prescription”.* I had never been exposed to a service that actively completed research within a cardiac rehabilitation setting, Lauren continued;

`*…a couple of years ago the HIIT (high intensity interval training) or MISS (moderate intensity steady-state training) trial was done here…it heavily influenced our exercise prescription…recently we`ve completed the SCAR trial with similar changes being made in how we exercise patients*.`

Interestingly, the research, although focused on exercise prescription in most cases also influenced MDT practice as a whole as Tom explained during our interview;

*Tom: “SCAR was a really big learning curve for the nursing team who were set in their ways with regards to enrolling patients, but we embraced it and changed our referral policies… implementation into wider practice, regional or beyond is hard though as it requires a change in resources and working practice”.*

Research was a driving force throughout this service epitomised by the working practice changes based on the scientific evidence and forward thinking. Whether it related to exercise prescription design or delivery, referral pathways, internal training programmes or progressive recognition of skills, this service pushed the boundaries in the field of cardiac rehabilitation through a determination to expand the evidence base and implement new findings into practice.

*When I reflected on what I had been told, I concluded that real-world practitioner research completed by highly qualified and skilled practitioners with academic understanding was the ideal solution for advancing the field. This service is unique due to its long standing relationship with the local university developed by practitioner links and the academic development of staff. Such collaboration demonstrates the value in closing the gaps between research and practice yet I had not experienced this before. The evidence they unearthed was shared within the team and the service adapted, it didn`t conform to traditional guidelines, they took the proactive approach to develop their own safe and more effective practice based on scientific literature which I feel can only be commended.*

#### CFIR 4.1 – theoretical knowledge levels

Exercise testing, assessment, interpretation, prescription, delivery and outcomes evaluation for individuals with chronic and complex conditions requires a specialist knowledge base and expertise [1, 3, 40]. Eight weeks into my visit and during an afternoon gym session I saw something that I had never seen in this setting before. Not unusually, patients were using their programme cards as guidance and referring to the charts on the walls on occasion for assistance in clarifying exercises or intensity, which answered one of my previous questions about Mira actively using resources rather than seeing them as decorations. But, more significantly as I walked past the rowing machines I noticed the speed and intensity of one particular patient. I know from speaking to Lauren that patient autonomy was encouraged, i.e., CEPs wanted patient`s to challenge themselves (safely) using the guidelines they have devised. Yet, this patient could have been in a regular `mainstream` gym. Rowing at a pace of ~ 2:00 min/500 meters for 1000 m is not something you generally see in a phase III cardiac setting in my experience, and would be challenging for most people. Technique was good; legs and arms in tandem, breathing maintained and visibly working hard. It was enlightening and I wanted to understand what gave the CEPs the confidence to safely prescribe and monitor this level of exercise and to have the confidence to let patients do it;

*Sam: “…core knowledge is physiology of the body, cardiovascular response to exercise and the cardiovascular disease process including risk factors…you definitely need to have done a bachelors in sport and exercise science…also a masters specifically in exercise physiology would be ideal covering associated pathologies like obesity, diabetes…we get a wide range of patients presenting with multiple morbidities”.*

Lauren reiterated the need for higher level academic qualifications and knowledge in exercise prescription and physiology, whilst stressing that experience in the role was vital. The stipulation of high-level qualifications (e.g., master`s degrees) is recommended in clinical exercise services [1, 3, 41] and in this service, it allowed for a more expansive patient inclusion/exclusion criteria as I discovered in one conversation with Tom;

*“Some services are much more cautious in terms of high-risk patients or exercising patients to a level that`s effective…we’ve done a lot of research and have staff that are highly qualified, so we accept higher risk conditions and understand how to safely progress them”.*

Academic knowledge of physiology and exercise prescription not only underpinned the way CEPs approached each patient but allowed a higher catchment of patients. Having AHCS-registered CEPs with exercise-specific knowledge and the application of it within research studies extended into challenging their own service effectiveness, alongside national guidelines which have been previously identified as ineffective [42–44].

*Discussions concerning current practice were frequent. The topic of other services not aligning with newly updated literature and that national guidelines were outdated, or least needed more depth, demonstrated that a culture of learning and service evolvement was in place. This service went further and applied changes based on the evidence base (some of which they created) to support their rationale for innovative exercise design and delivery.*

#### CFIR Sect. 4.2 – practical skill application and competency (effective task completion)

Clinical exercise skills relate to the practical application of theoretical learning (e.g., conducting physical assessments) and the ability to communicate the information effectively to patients [1, 45]. Having previously discussed the importance of monitoring physiological responses in ECG or blood pressure during assessment, the implementation of the latest HIIT exercise design and acceptance of complex (higher risk) patients, it became clear that monitoring patients was a critical skill. One area of this was the ability to demonstrate and identify how to progress (or regress) exercise by coaching patients through sessions and leading group exercise activities (warm up/cool down) [46] as explained by Lauren after a group session warm up;

*Lauren: “…putting exercise onto paper is the theory behind the practice….coaching patients, leading the class, making sure that everybody is safe and they understand what you`re asking them to do are the practical skills….ongoing monitoring using RPE, heart rate and pain scales is vital…adaptations could be range of movement, intensity, options for balance, avoiding exercises that could exacerbate risk such as quick turns or direction changes…we have to respond to the patient, how they feel…it cannot be generic and it might change daily”.* 

*Working patients intermittently at heart rate percentages challenging 70% or above required clinical knowledge of physiology, patient history, accurate fitness assessments, precise exercise prescription and most importantly, excellent exercise delivery skills and monitoring throughout. For me, such replication across services requires an AHCS-CEP level of knowledge and skills as those individuals have demonstrated competence in adapting exercise to meet the needs of each individual and are capable of monitoring them at a level that minimises risk yet increase effectiveness.*

The CEP role is diverse and complex, therefore, skills and competencies are wide ranging and not just associated with exercise per se [1]. Tom expanded and emphasised that potential employees (in this service) needed communication skills such as empathy, but also experience (and competency) in working with real patients;

*Tom: “…we have some really challenging patients that don’t want to engage or haven’t got the literacy levels…CEPs need some of those softer skills that help to deliver personalized care to patients. I feel that at the moment that isn’t there nationally, when we’re interviewing CEPs with academic ability (master’s level) they don’t know what they don’t know… they’re hit with patients that come from all sorts of backgrounds, have all sorts of challenges and this is where soft skills come in”.*

The combination of CEP and wider MDT interaction during daily/weekly huddles, the learning culture of shared practice and craft learning (teachable moments), alongside research exposure and proficiency in communication ensures that the knowledge and skills of this team were exceptional. I feel that this combination of theoretical and practical learning sees knowledge, skills and competency unify [22, 40]. Similar research [40] identified that education alone would not be sufficient to create a well-rounded or complete CEP. The specialist nature of the role requires exposure to real world practice, with peer support and training (akin to Tom`s suggestions) essential [38, 46]. This service provided that support network in abundance with staff able to reference it when applying for AHCS registration.

*Hearing Tom, Lauren and Sam discuss the knowledge, skills and competencies required by a CEP made me think about how the AHCS registration could change the landscape of clinical exercise provision. Firstly, individuals could demonstrate they had undertaken the education and training akin to other health care professionals (e.g., physiotherapists), including rigorous assessments and exposure to the up-to-date scientific evidence base. Secondly, academic institutions would have to conform to Clinical Exercise Physiology UK standards to gain AHCS accreditation, updating their curriculum accordingly and ensuring it contained suitable work placements for students to observe those teachable moments (one of Tom`s concerns). Finally, I know from my own registration that behaviour change and communication skills feature prominently within the registration requirements, thus increasing student proficiency in these areas is essential for effective service provision. This service is unique, it has multiple AHCS-registered CEPs within it and a research arm that provides opportunities for staff to participate in innovative projects that undoubtably advance their knowledge within the field. Even so, a learning culture that supports staff and enhances their skills through shared practice has been created and exposure to it makes me feel that we (clinical exercise service providers) have to learn from it.*

#### CFIR Sect. 5.1 - service effectiveness

My experience has shown me that service effectiveness can be subjective depending on who you are talking to and what level of interest (or bias) they have. Translating evidence into practice is not always easy, it requires a high level of understanding between team members who can communicate with each other to implement change as described by Lauren;

*Lauren: “…we`ve all got different ways of prescribing exercise but we have a similar level of education, so if I talk about something and explain what I’m doing the other person understands…so qualifications are a big part of it (effectiveness) plus this culture of always trying to find the best ways of working*…*the passion of the staff that genuinely want to give their best and as a result of that our programme has got lots of options (for patients)”.*

Moreover, the individualised case management approach, identified as effective in clinical MDT settings [37], was one of the real strengths of the service displaying a dedication to patient-centred care as recognised by Tom: *“.from bedside to cardiac discharge post-exercise the patient receives personalised care…we help people get back to what they want to do in the long-term”.* It would be remis of me not to highlight the strict NHS safety protocols in place which ensured streamlined referral pathways and the safeguarding of patients throughout their journey. Additionally, the facilities that were available to patients were, in my opinion, unrivalled in this field and duly recognised by the team while we were delivering exercise sessions;

“…w*e are lucky with what we have in terms of our facilities…we’re able to use a fully equipped gym…we have consultation rooms, assessment rooms with dedicated equipment and clinical monitoring, other services only have circuit-based portable equipment”* (Sam).

These processes all contributed to high levels of patient care highlighted by the 81% uptake from those eligible into the service, compared to the national average of 52% [7].

### Strengths and limitations

The main strength of this study was the multi-method exploration of clinical exercise provision which allowed an in depth look at service operations and effectiveness, including staff knowledge, skills, competencies and challenges. The study was however conducted as a single service case study focusing on one long-term condition, therefore limited by a small, convenience sample. Some caution must be taken in generalising across the cardiac rehabilitation landscape as this service, although operating as an MDT, solely employed CEPs for exercise provision and had links to an academic institution for the research activities which is not widely available in the UK. It does, however, go some way in explaining the key considerations for effective clinical exercise provision for a long-term condition.

## Conclusion

AHCS-registered CEPs within this cardiac-specific, hospital and community-based service were essential for the provision of innovative and individualised exercise prescription, underpinned by their high levels of academic education and participation in real-world clinical research trials. The localised (site specific) MDT structure enabled staff upskilling through shared peer experiences, observations and collaborative working between CEPs and healthcare professionals, ensuring effective working practices were maintained throughout the service. Clinical nurse specialists enabled a smooth transition of referrals from hospital into the exercise component of the service and delivered most of the behaviour change elements of the programme. Registered CEPs were able to take part in impromptu lifestyle conversations and `teachable moments` with patients, yet a clear strategy for delivering behaviour change information required development. It is important to consider that this cardiac-specific service was unique by solely employing registered CEPs for exercise provision and had links to an academic institution for the research activities which is not widely available in the UK. Future research should explore daily practices within CEP-led clinical exercise services across other long-term conditions (e.g., pulmonary rehabilitation) to assist in the generalisation of findings. These observations should focus on understanding how exercise specialists utilise their knowledge, skills and competencies within a service to create optimal exercise prescription.

### Supplementary Information

Additional file 1. Interview guides cited in text are provided for staff participants and patients.

## Data Availability

The datasets used and / or analyzed during the current study are available from the corresponding author on reasonable request.

## Abbreviations

BACPR: British Association for Cardiovascular Prevention and Rehabilitation
CEP: Clinical Exercise Physiologist
ECG: Electrocardiogram
MDT: Multi Disciplinary Team
NACR: National Audit of Cardiac Rehabilitation
NHS: National Health Service
AHCS: Academy for Healthcare Science
TIDieR: Template for Intervention Description and Replication
