# Supplementary material for: A multi-method exploration of a cardiac rehabilitation service delivered by registered Clinical Exercise Physiologists in the UK: key learnings for current and new services
Source: BMC Sports Sci Med Rehabil. 2024 Jun 8;16:127. doi: 10.1186/s13102-024-00907-4 (PMC11162017; doi:10.1186/s13102-024-00907-4)
Supplement: Supplementary file 1 — Additional file 1. Interview guides cited in text are provided for staff participants and patients. [file 13102_2024_907_MOESM1_ESM.docx]

**Additional file 1: Interview / Focus groups guide**

**Introduction**

The interview / focus group will last approximately 20-90 minutes and will explore 3-5 main areas (depending on which group you are in); intervention design and evidence, patient needs and resources, structural characteristics of the organization, knowledge and beliefs and planning.

I would like to remind you that the interview / focus group will be audio recorded. The audio recording is essential to your participation but you should be comfortable with the audio recording process. Therefore, you are free to stop the audio recording at any time and therefore withdraw your participation.

| **CFIR Domain** | **CFIR areas to consider** | **Overarching interview question (*prompts and probes will follow based on answers)** |
| --- | --- | --- |
| **Intervention development & challenges** | **Intervention design & evidence** | How was the intervention developed: what were the challenges that needed to be overcome and how was this achieved? |
| **Service users and resources** | **Patient needs & resources** | What barriers do the service users face to participating in the intervention and what resources are available to support them? (If any, please provide examples) |
| **Organisation & structures** | **Structural characteristics** | How does the structure of your service (staffing, age, size, etc.) impact the implementation of the intervention? |
| **Staff skills & perspectives** | **Knowledge and beliefs** | What level of qualifications, skills and competencies do you feel are required to work within this service and make it effective?  How does the organizational staff structure foster effective delivery of exercise?  What challenges exist in delivering coherent, high quality exercise provision and how are they overcome using existing staff resources? |
| **Service process and effectiveness** | **Planning** | Can you describe how and why your feel this service is / is not effective? |

Thank you, that’s the end of my questions. To finish off, could I summarize my understanding of intervention design and evidence, patient needs and resources, structural characteristics of the organization, knowledge and beliefs and planning (summarize key points depending on which components have been discussed)…..have I understood your views correctly or is there anything you would like to add?

**If no**

Thank and finish interview.

*Prompts/Probes are there to be used as a guide for the interviewer. They are flexible questions to try and elicit responses from the participant. They should be used in a conversational manner and only when deemed appropriate.
